# Withdrawal/Withholding of Life-Sustaining Therapies: Limitation of Therapeutic Effort in the Intensive Care Unit

**DOI:** 10.3390/medicina60091461

**Published:** 2024-09-06

**Authors:** Ángel Becerra-Bolaños, Daniela F. Ramos-Ahumada, Lorena Herrera-Rodríguez, Lucía Valencia-Sola, Nazario Ojeda-Betancor, Aurelio Rodríguez-Pérez

**Affiliations:** 1 Department of Anesthesiology, Intensive Care and Pain Medicine, Hospital Universitario de Gran Canaria Doctor Negrín, 35010 Las Palmas de Gran Canaria, Spain; lorehe8@gmail.com (L.H.-R.); ori98es@yahoo.es (L.V.-S.); nojebet@gobiernodecanarias.org (N.O.-B.); arodperp@gobiernodecanarias.org (A.R.-P.); 2 Department of Medical and Surgical Sciences, Universidad de Las Palmas de Gran Canaria, 35001 Las Palmas de Gran Canaria, Spain; daniela_ramos_ahumada@hotmail.com

**Keywords:** intensive care unit, life-sustaining therapies, futility, withholding, withdrawal, end-of-life

## Abstract

*Background/Objectives*: The change in critically ill patients makes limitation of therapeutic effort (LTE) a widespread practice when therapeutic goals cannot be achieved. We aimed to describe the application of LTE in a post-surgical Intensive Care Unit (ICU), analyze the measures used, the characteristics of the patients, and their evolution. *Methods:* Retrospective observational study, including all patients to whom LTE was applied in a postsurgical ICU between January 2021 and December 2022. The LTE defined were brain death, withdrawal of measures, and withholding. Withholding limitations included orders for no cardiopulmonary resuscitation, no orotracheal intubation, no reintubation, no tracheostomy, no renal replacement therapies, and no vasoactive support. Patient and ICU admission data were related to the applied LTE. *Results*: Of the 2056 admitted, LTE protocols were applied to 106 patients. The prevalence of LTE in the ICU was 5.1%. Data were analyzed in 80 patients. A total of 91.2% of patients had been admitted in an emergency situation, and 56.2% had been admitted after surgery. The most widespread limitation was treatment withholding (83.8%) compared to withdrawal (13.8%). No differences were found regarding who made the decision and the type of limitation employed. However, patients with the limitation of no intubation had a longer stay (*p* = 0.025). Additionally, the order of not starting or increasing vasopressor support resulted in a longer hospital stay (*p* = 0.007) and a significantly longer stay until death (*p* = 0.044). *Conclusions*: LTE is a frequent measure in critically ill patient management and is less common in the postoperative setting. The most widespread measure was withholding, with the do-not-resuscitate order being the most common. The decision was made mainly by the medical team and the family, respecting the wishes of the patients. A joint patient-centered approach should be made in these decisions to avoid futile treatment and ensure end-of-life comfort.

## 1. Introduction

Progress in both society and healthcare has led to a new profile of patients with a longer life expectancy and more comorbidities [1]. Also, cognitive and technological advances have made it possible to substitute for vital functions efficiently in critical situations, directing therapeutic efforts for the treatment of decompensations of chronic processes [2]. The main objective of critical care is to support patients, compensating for failing vital functions while they are in danger. It is a balance between “doing everything medically possible” and “assuring the patient does not suffer” [3]. The growth of intensive care units (ICU) has made the management of critically ill patients more efficient, leading to prolonging their lives in many cases [4]. However, since the baseline patient situation is not always optimal, the extent to which it is appropriate to continue treatment should be considered, as well as avoiding therapeutic obstinacy. Treatment should be focused on the benefit and quality of life in accordance with the bioethical principles of autonomy, beneficence, justice and non-maleficence [5]. There is wide variation in the bioethical principles knowledge and their application to resolve ethical conflicts in the ICU [6]. In addition, discrepancies might appear among professionals in estimating the prognosis of critical patients based solely on patients’ quality of life [7]. Thus, efforts must be made to consider every patient as a unique individual [8], taking into account the principle of dignity also in the process of adjusting treatments. As the application of futile treatments has been shown to endanger the maintenance of patients’ dignity [9], the term “dignity” has been introduced into the different definitions of medical futility [10], which may conflict with the concept of “quality of life”. Therefore, dignity must be addressed during patients’ management, even more so in the intensive care context [11].

Limitation of therapeutic effort (LTE) includes both withholding and withdrawal of life support in patients since the outcome is not expected to outweigh the benefits of not implementing them [2]. Limiting therapeutic effort can be carried out by withdrawing the established treatment (vasoactive support, renal replacement therapies or mechanical ventilation, among others) or by therapeutic abstention, by which a therapeutic “limit” is established and more aggressive measures or the increase in those already established is restricted. This approach to medicine is particularly important for critically ill patients to avoid therapeutic futility [11]. This measure usually involves the clinical judgment of the medical team along with the patient or family members if the patient is unable to make decisions [12]. Family members cannot evaluate the extent to which critically ill patients experience distress [13]. As the decision-making process in critically ill patients might be complex, it has been recommended that institutions have strategies to prevent conflicts that may arise between healthcare professionals regarding the decision of whether a treatment is appropriate or not [14].

The profile change that we are currently facing in our patients makes LTE a widespread practice worldwide when it is not possible to achieve the therapeutic goal [15]. Despite the expansion of LTE in the ICU, the main determinants are still unknown. Clinical judgment concerning the futility of a measure is difficult since, in the clinical setting, there are usually no absolute certainties about a patient’s prognosis [16]. The prognosis of the pathology that led to the patient’s admission to the ICU is not the only criterion for establishing a therapeutic prognosis. In addition to physiological futility, in which the measures are not capable of reaching the intended goal, there are other quantitative or qualitative criteria for discontinuing treatment. Quantitative criteria are based on scoring systems that establish a numerical standard that determines that a treatment is inappropriate; on the other hand, qualitative criteria are based on the balance between benefits and harms [17]. Furthermore, there are occasions when any type of therapy is futile, as in cases of brain death, where continuing any type of life support constitutes a medical error. Therefore, a multitude of criteria must be considered to accurately assess the therapeutic prospects in the context of the critically ill patient. However, the main impediment to LTE is still the uncertainty of the outcome of a patient with the possibility of condemning him/her to an inescapable death. The aim of this study was to describe the characteristics of patients and their admission that led to the decision to initiate LTE measures in a postsurgical ICU. The type of LTE most frequently used and patients’ evolution after LTE instauration were also evaluated.

## 2. Materials and Methods

After the approval of the Ethics Committee of the Hospital Universitario de Gran Canaria Doctor Negrín was obtained (code 2023-028-1, approved on 1 February 2023), a descriptive observational retrospective study of a prospectively filled database was carried out. The data had been prospectively collected in a protected database after being anonymized. Thus, when analyzing the data, patients could not be identified. This analysis was performed by investigators who were not involved in the data collection or in completing the database. Of all patients admitted to the postsurgical ICU between January 2021 and December 2022, those to whom LTE was applied were included. Patients without complete information and those whose histories did not include data about LTE were excluded. The manuscript follows the STROBE guidelines [18].

### 2.1. Study Variables

The following LTE circumstances were defined for this study: brain death, withdrawal of measures, and therapeutic abstinence (withholding). Withholding included the following: orders for no cardiopulmonary resuscitation, no orotracheal intubation, no reintubation, no tracheostomy, no renal replacement therapies, and no vasoactive support. The information collected on the characteristics of LTE were the following: type of LTE and who made the LTE proposal, whether by Advance Directives, family, medical staff, or joint decision.

The database of the study was composed of variables extracted from the electronic medical records of the patients selected. Age, sex, height, weight and body mass index (BMI) were recorded, as well as the following comorbidities: ischemic heart disease, heart failure, valvular heart disease, arterial hypertension, chronic obstructive pulmonary disease (COPD), diabetes mellitus, dyslipidemia, peripheral vascular disease, cerebrovascular disease, hemiplegia, chronic kidney disease, liver pathology, neoplasia, leukemia, lymphoma, metastasis, connective tissue pathology, ulcers, AIDS, dementia, previous surgical interventions and smoking. Then, the Charlson Comorbidity Index was calculated using the comorbidities collected.

Regarding the characteristics of the patient’s admission, a distinction was made between medical or surgical admissions and urgent or programmed admissions. Likewise, the values of the last blood test prior to the establishment of LTE were obtained, such as hemoglobin, creatinine, C-reactive protein, procalcitonin, leukocytes, platelets and lactic acid. Hospital stay prior to admission to the postsurgical ICU, hospital stay in this unit and hospital stay after discharge from the unit were also recorded, as well as complications during admission, such as acute renal failure, surgical wound infection, postoperative bleeding, agitation and cardiorespiratory arrest. Finally, the maximum and last respiratory, hemodynamic and renal support measures prior to the establishment of LTE, the outcome and whether comfort measures were applied were recorded.

### 2.2. Statistical Analysis

The data were analyzed using SPSS v.24.0 (Statistical Package for Social Sciences, IBM, Chicago, IL, USA). Quantitative variables were expressed as mean ± SD if the distribution was normal or as median and interquartile range (25th and 75th percentiles) if the distribution did not adjust to normality. The Shapiro–Wilk test was used to check the normality of the data. To compare means, Student’s *t*-test was used if the comparison was between two groups or analysis of variance (ANOVA) if the comparison was among more than two groups. Qualitative variables were expressed as absolute and relative frequencies. The chi-square test was used for comparison among different groups. A *p* < 0.05 was established as the level of statistical significance.

## 3. Results

**Figure 1:**
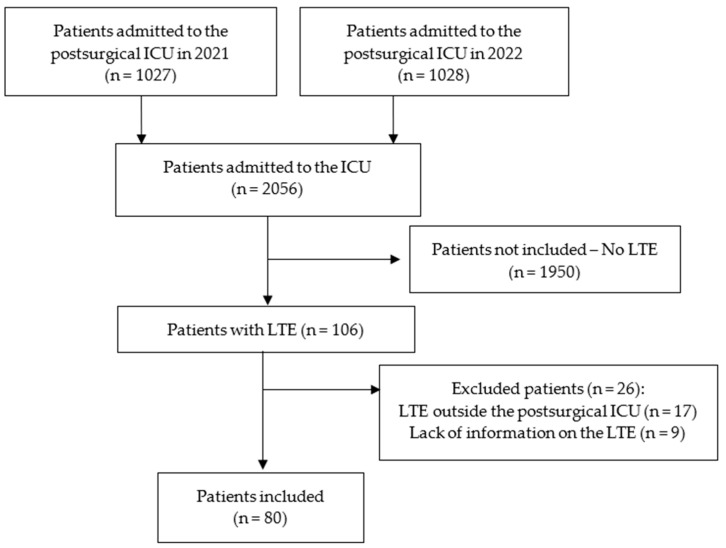
Flow diagram of patients included in the analysis.

**Figure 2:**
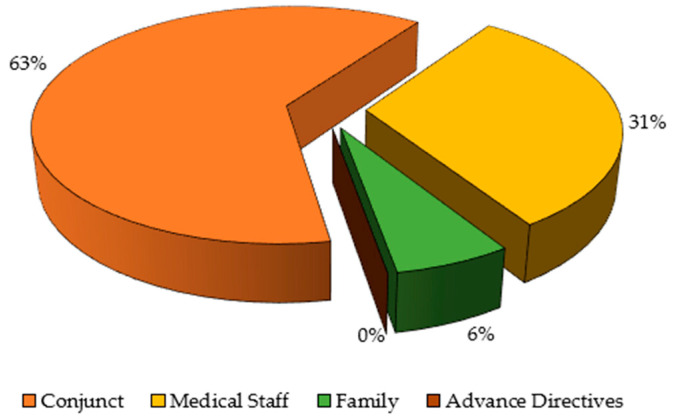
Limitation of therapeutic effort decision proposal.

During the study period, 2055 patients were admitted to the postsurgical ICU (1027 patients in 2021 and 1028 in 2022). Of those, the therapeutic effort was limited to 106 patients (5.1%). After applying the exclusion criteria, those without complete information or those to whom LTE was not applied in the postsurgical ICU, the data from 80 patients were analyzed (Figure 1). Patients and admission characteristics are shown in Table 1. In this surgical ICU of a tertiary university hospital, many patients were admitted for medical reasons due to the lack of beds in other medical ICUs of the hospital. In addition, patients show multiple comorbidities and are frequently operated on for complex pathologies/surgeries, requiring a prolonged postoperative stay in the ICU. Figure 2 and Table 2 show the LTE characteristics. Of the patients analyzed, 91.2% had been admitted for an emergency, and 56.2% had been admitted after surgery.

No statistically significant relationship was found between the type of limitation applied and who made the decision (*p* = 0.056) or if the patient was admitted after surgery or after a non-surgical reason (*p* = 0.373). Withholding was performed in 87.7% of patients admitted for an emergency and in 42.8% of those admitted according to a schedule (*p* = 0.005). No patients admitted with COVID-19 underwent withdrawal as part of LTE, but no statistical differences were found among the applied LTE in these patients (*p* = 0.222). Table 3 and Table 4 show the differences among the groups and the type of LTE.

The different types of withholding applied were analyzed. No differences were found concerning the time between admission to the ICU and the LTE decision according to the order for non-cardiopulmonary resuscitation (*p* = 0.501), non-renal replacement (*p* = 0.866), no vasoactive support therapies (*p* = 0.126) or no reintubation (*p* = 0.665). When the therapeutic ceiling did not allow for tracheostomy, the LTE decision was made earlier (6.3 ± 7.1 days vs. 18.3 ± 19.8 days, *p* = 0.008). In addition, patients who were refused a tracheostomy stayed longer in the ICU than those who were not (7.6 ± 7.2 days vs. 20.2 ± 21.8 days, *p* = 0.008). In patients with an order of no orotracheal intubation, LTE was decided before (4.6 ± 4.1 days vs. 11.9 ± 15.1 days, *p* = 0.028) and survived longer before death (5.7 ± 8.8 days vs. 2.4 ± 4.3 days, *p* = 0.025) than those patients for whom therapeutic limitation did not prevent orotracheal intubation.

## 4. Discussion

This retrospective study shows the routine clinical practice regarding LTE in a postsurgical ICU, which was applied to 5.1% of the patients. This percentage is lower than stated in previous prospective studies, ranging from 12% in a prospective study performed in 2007 [2] to 11.8% in a multicenter study published in 2022 [19]. However, a retrospective study performed on 1603 patients admitted to a Portuguese ICU detected an incidence of LTE similar to ours (7.6%) [20]. The population included in our sample is mostly surgical, and the decision of LTE is more frequent in medical ICUs [2]. Thus, a retrospective study involving septic patients admitted to a medical ICU detected a rate of 36.5%, with a Charlson Comorbidity Index similar to that of our population [21]. Charlson Comorbidity Index was not taken into consideration in the sample when LTE was decided but was calculated for this analysis, showing a relatively low 10-year survival. Likewise, most patients had been admitted urgently and were older than 70 years, at higher risk of LTE during ICU admission [20,21].

The type of LTE most employed in our population was withholding, as in other studies [2]. Although withdrawal was performed in a higher proportion in a multicenter study (45%) [19], the decision to withdraw measures is perceived as more aggressive since it implies the removal of support, leading to death. Within withholding treatment, the most frequently used measure in our patients was the order of do not resuscitate (DNR), followed by not performing tracheostomy. Patients and families may have a more serious concept of these measures than renal replacement therapies and vasoactive support. Studies exploring the application of therapeutic restraint focus on non-cardiopulmonary resuscitation [22]. This measure was highly supported during the COVID-19 pandemic because cardiorespiratory arrest in COVID-19 patients occurred in asystole, entailing a worse prognosis at a time when the demand for care exceeded the available resources [23]. On the other hand, the limitation of administering vasoactive support or extrarenal depuration therapies may be less familiar or sound less invasive for patients. Currently, refusal or discontinuation of dialysis in elderly patients are generally practices that are well accepted by many physicians, even in spite of patient rejection [24]. Despite the high mortality of not using extrarenal depuration therapies, their inhibition is well tolerated as these techniques are associated with a decrease in quality of life [25]. The limitations of non-intubation or non-reintubation are relegated to the last positions of the measures adopted in our population since most postoperative patients were already intubated upon admission.

Considering LTE decisions in advance reduces anxiety and stress for both patients and families [26]. Discussion of LTE during the first 48 h of admission avoids procedures considered futile and diminishes the perception of loss of dignity or suffering [27] without increasing patient mortality, compared to discussing it later during the ICU admission [26]. The decision, in most cases, was made by the medical team together with the family. Joint decision-making between the treating physicians and the family is desirable [28], involving the active participation of family members, who play a fundamental role when the patient is not autonomous [29]. However, the decision to withdraw supportive measures can be stressful and cause a moral dilemma for caregivers who are responsible for removing all life-supporting devices and treatments [30,31]. A qualitative study carried out on experienced ICU nurses who had been involved in LTE implementation showed that they were relieved when employing these measures, although they acknowledged that there was an imbalance between medical and nursing staff in the decision-making process [32]. In addition, collaboration among different professionals treating the patient has been shown to improve patient care, decreasing the occurrence of complications and enhancing trust and communication with patients and staff [33]. A study published in 2001 showed that the LTE proposal came from the medical staff in 92.9% of LTE cases, and families were not involved in the decision-making process in 28.3% of the cases [28]. Only 9.3% of LTE patients had expressed a desire to refuse life-prolonging therapies prior to ICU admission [28]. There has been an increase in information about patients’ wishes and a greater involvement of patients and family members in decision-making over the years [34], facilitating greater consistency with the bioethical principle of patient autonomy, despite the clinical situation that prevents active decision making. However, the lack of Advance Directives in all our cases indicates a lack of awareness in society regarding personal decision making in critical situations [35]. Advance Directives in other populations with a better short-term prognosis have been shown to be unstable, with multiple changes over time [36]. In fact, DNR orders are usually postponed until imminent death instead of early placement [37], leading families to difficult situations. A study conducted on 257 individuals (94 patients and 163 relatives) exploring their perception of LTE in the ICU showed that 60% of participants would agree to apply LTE in cases of poor prognosis, regardless of altered quality of life [35]. The main reasons for proposing LTE in the ICU are poor prognosis, potential suffering, or the expectation of poor quality of life after admission [38]. With the intention of avoiding futile therapies, different mortality scales used today in critical care units, such as APACHE II (Acute Physiology and Chronic Health Evaluation II) [2,17,21,22], SOFA (Sequential Organ Failure Assessment) [2,26], SAPS II (Simplified Acute Physiology Score II) [20] or Charlson Comorbidity Index [21,27], are helpful as predictors of mortality and have been shown to be good indicators for which patients should undergo LTE. Other scales have been developed to predict the probability of survival with good neurological outcomes after cardiac arrest. However, predictions made by these scales do not always correlate with predictions made by clinicians [39]. So, it might be interesting to perform a comprehensive assessment prior to admission to obtain an overview of a patient’s situation and properly assess the risks and benefits of our therapeutic efforts [40].

In instances of unfavorable outcomes, all patients in our postsurgical ICU received sedation, thanks to the anesthesiologists’ expertise in pain management and comfort measures. It underlines the importance of our accompaniment in the end-of-life process in a dignified way, without pain or suffering [37]. Mortality associated with LTE is variable (between 40% and 90%), depending on the characteristics of the hospital and the population analyzed [40]. In a large percentage of the patients in our sample, death occurred in the unit (72.5%) or during hospital admission (93.7%). A study conducted more than 20 years ago detected similar mortalities to those of our study (69% in the ICU and 91% during hospital admission) and a similar survival at one year (4%) [2]. Nevertheless, five of the patients who underwent LTE were discharged and completed 1 year of survival after admission. Thus, treatment adequacy does not always lead to a patient’s death. Our efforts as professionals should not end when the decision is made to limit treatment for a patient [41], but rather, we should increase them to obtain the best possible results while avoiding suffering.

### Limitations

Since this is a retrospective study, the main limitation was the loss of data or patients. However, patients were collected from a prospectively filled database, and data were registered as exhaustively as possible in the electronic medical records. Although information from 80 out of 106 patients could be retrieved, the absence of statistical significance in many of the associations may be due to the lack of strength of the study. However, it fulfills the proposed objective of representing LTE in patients admitted for two years to a postsurgical ICU of a tertiary university hospital. Another limitation of this research has been the scarcity of recently published studies on the application of withdrawal/withholding measures in postsurgical ICUs that would allow us to compare the data obtained with other similar populations.

## 5. Conclusions

LTE is frequently employed in the ICU, although, to a lesser extent, it is employed in the postsurgical setting. The most widespread measure is therapeutic abstention (withholding), with the DNR order being the most common practice, followed by no tracheostomy, no renal support and no vasoactive drugs. The approach to the critically ill patient must be comprehensive, respecting his/her wishes and avoiding therapeutic futility, and the decision to employ LTE measures should take this into account. Therefore, caregivers should learn the patient’s wishes as soon as possible. To strengthen the ethical aspects of critically ill patients, all the information related to LTE in this population should be routinely documented.

## Figures and Tables

**Table 1:** Characteristics of patients to whom limitation of therapeutic effort was applied and their admission.

		n = 80
Sex male, n (%)		48 (60.0)
Age, years		74 ± 12
BMI, kg·m^−2^		25.8 ± 5.5
Comorbidities, n (%)	Arterial hypertension	59 (73.8)
	Dyslipidemia	48 (60.0)
	Diabetes mellitus	31 (38.8)
	End-stage renal disease	21 (26.3)
	Ischemic Cardiopathy	20 (25.0)
	Heart failure	17 (21.3)
	Cancer	15 (18.8)
	Liver disease	14 (17.5)
	COPD	12 (15.0)
	Peripheral vascular disease	11 (13.8)
	Atrial fibrillation	10 (12.5)
	Active Smoking	8 (10.0)
	Dementia	6 (7.5)
Charlson Comorbidity Index		5.75 ± 2.43
Estimated survival at 10 years according to Charlson Comorbidity Index, %		24.7 ± 32.7
ASA III, n (%)		48 (60.0)
COVID-19 Infection, n (%)		13 (16.3)
Length of stay, days	In the ward, prior to the UCI	8.1 ± 17.2
	In the ICU	11.4 ± 13.9
Department of origin, n (%)	General and Digestive Surgery	30 (37.5)
	Internal Medicine	16 (20.0)
	Vascular Surgery	5 (6.3)
	Orthopedic and Traumatology	4 (5.0)
	Neurosurgery	4 (5.0)
	Cardiology	4 (5.0)
	Others	17 (21.3)
Complications during admission to the ICU, n (%)	Acute Renal Failure	62 (77.5)
	Agitation	24 (30.0)
	Surgical wound infection	20 (25.0)
	Postoperative bleeding	8 (10.0)
	Cardiorespiratory arrest	7 (8.8)
Maximum respiratory support measures, n (%)	Tracheostomy	15 (18.8)
	Orotracheal intubation	47 (58.8)
	High-flow nasal canula	11 (13.8)
	BiPAP	11 (13.8)
Maximum hemodynamic support, n (%): Vasoactive support		71 (88.8)
Maximum measures of renal support, n (%)	Furosemide in continuous infusion	34 (42.5)
	Extrarenal depuration therapy	30 (37.5)

Data are expressed as absolute and relative frequencies or mean ± SD. BMI: Body mass index; COPD: Chronic obstructive pulmonary disease; ASA: American Society of Anesthesiologists physical status; ICU: intensive care unit; BiPAP: bilevel positive airway pressure.

**Table 2:** Characteristics of the limitation of therapeutic effort applied.

		n = 80
Time between admission to the ICU and initiation of LET, days		9.9 ± 13.4
Type of LTE, n (%)	**Withholding**	**67 (83.8)**
	No-cardiopulmonary resuscitation	67 (83.8)
	No tracheostomy	56 (70.0)
	No renal replacement therapies	44 (55.0)
	No vasoactive support	29 (36.2)
	No orotracheal intubation	22 (27.5)
	No re-intubation	7 (8.8)
	**Withdrawal**	**11 (13.8)**
	**Brain death**	**2 (2.5)**
Final respiratory support measures, n (%)	Tracheostomy	14 (17.5)
	Orotracheal intubation	38 (47.5)
	High-flow nasal canula	11 (13.8)
	BiPAP	7 (8.8)
Final hemodynamic support measures, n (%): Vasoactive support		47 (58.8)
Final renal support measures, n (%)	Furosemide in continuous infusion	27 (33.8)
	Extrarenal depuration therapy	24 (30.0)
Outcome of LTE, n (%)	Death in the ICU	58 (72.5)
	Death in Hospital	75 (93.7)
	Survival at one year	5 (6.3)
Time between LTE initiation and death, days		3.3 ± 6.0

Data are expressed as absolute and relative frequencies or mean ± SD. LTE: limitation of therapeutic effort; ICU: intensive care unit; BiPAP: bilevel positive airway pressure.

**Table 3:** Type of limitation of therapeutic effort applied and patients’ characteristics prior to admission to the postsurgical ICU.

		Withdrawal (n = 11)	Withholding (n = 67)	Brain Death (n = 2)	*p*
Hospital stay prior to admission to the ICU, days		5.8 ± 8.7	8.7 ± 18.4	0.0	0.703
Sex male, n (%)		5 (45.4)	43 (64.2)	0 (0.0)	0.108
Age, years		67 ± 21	76 ± 9	49 + 9	0.001
Comorbidities, n (%)	Arterial hypertension	4 (36.4)	54 (80.6)	1 (50.0)	0.006
	Dyslipidemia	3 (27.3)	44 (65.6)	1 (50.0)	0.053
	Diabetes mellitus	2 (18.2)	29 (43.0)	0 (0.0)	0.149
	End-stage renal disease	2 (18,2)	18 (26.9)	1 (50.0)	0.617
	Ischemic cardiopathy	2 (18.2)	18 (26.9)	0 (0.0)	0.587
	Heart failure	1 (9.1)	16 (23.9)	0 (0.0)	0.409
	Cancer	2 (18.2)	13 (19.4)	0 (0.0)	0.786
	Liver disease	1 (9.1)	13 (19.4)	0 (0.0)	0.568
	COPD	2 (18.2)	10 (14.9)	0 (0.0)	0.802
	Peripheral vascular disease	0 (0.0)	11 (16.4)	0 (0.0)	0.290
	Atrial Fibrillation	0 (0.0)	10 (14.9)	0 (0.0)	0.330
	Active Smoking	2 (18.2)	6 (8.9)	0 (0.0)	0.571
	Dementia	0 (0.0)	6 (8.9)	0 (0.0)	0.533
ASA III, n (%)		5 (45.4)	42 (62.7)	1 (50.0)	0.262
Charlson Comorbidity Index		4.2 ± 2.4	6.1 ± 2.2	1.5 ± 2.1	0.001

Data are expressed as absolute and relative frequencies or mean ± SD. ICU: intensive care unit; COPD: Chronic obstructive pulmonary disease; ASA: American Society of Anesthesiologists physical status.

**Table 4:** Type of limitation of therapeutic effort applied and variables related to the ICU admission.

		Withdrawal (n = 11)	Withholding (n = 67)	Brain Death (n = 2)	*p*
Stay in the postsurgical ICU, days		10.8 ± 11.1	11.8 ± 14.6	2.5 ± 0.7	0.650
Time between admission and initiation of LTE, days		9.3 ± 10.2	10.3 ± 14.1	1.5 ± 0.7	0.657
Time between LTE initiation and death, days		1.2 ± 1.7	3.7 ± 6.4	1.0 ± 0.0	0.378
Emergent admission, n (%)		8 (72.7)	64 (95.5)	1 (50.0)	0.005
Cause of admission, n (%)	Surgical	7 (63.6)	36 (53.7)	2 (100.0)	0.373
	Medical	4 (36.4)	31 (46.3)	0 (0.0)	
COVID-19 Infection, n (%)		0 (0.0)	13 (19.4)	0 (0.0)	0.222
Proposal of LTE, n (%)	Medical Team	1 (9.1)	22 (32.8)	2 (100.0)	0.056
	Family	2 (18.2)	3 (4.5)	0 (0.0)	
	Conjunct	8 (72.7)	42 (62.7)	0 (0.0)	
Hemoglobin, g·dL^−1^		9.8 ± 1.9	9.3 ± 1.9	9.6 ± 0.2	0.711
Creatinine, mg·dL^−1^		1.6 ± 1.4	1.9 ± 1.2	4.9 ± 2.9	0.004
C Reactive Protein, mg·L^−1^		145 ± 115	140 ± 101	89 ± 108	0.776
Procalcitonin, ng·mL^−1^		26.1 ± 45.6	22.5 ± 56.5	29.5 ± 0.3	0.979
Leukocytes, 10^3^·uL^−1^		19.7 ± 9.4	28.5 ± 76.1	25.2 ± 17.8	0.928
Lactic Acid, mmol·L^−1^		1.9 ± 1.2	3.2 ± 4.3	1.8 ± 1.1	0.544
Platelets, 10^3^·uL^−1^		235 ± 185	160 ± 105	173 ± 150	0.166
Complications during admission, n (%)	Acute Renal Failure	7 (63.6)	54 (80.6)	1 (50.0)	0.294
	Agitation	0 (0.0)	24 (35.8)	0 (0.0)	0.036
	Surgical wound infection	4 (36.4)	16 (23.9)	0 (0.0)	0.480
	Postoperative bleeding	5 (45.5)	3 (4.5)	0 (0.0)	<0.001
	Cardiorespiratory arrest	0 (0.0)	7 (10.4)	0 (0.0)	0.475

Data are expressed as absolute and relative frequencies or mean ± SD. ICU: intensive care unit.

## Data Availability

The data presented in this study are available upon request from the corresponding author.
